# Proteinuric kidney disease in children at Queen Elizabeth Central Hospital, Malawi

**DOI:** 10.1186/s12882-018-0832-6

**Published:** 2018-01-31

**Authors:** Zondiwe Victor Mwanza, Mignon McCulloch, Mark Drayson, Timothy Plant, David V. Milford, Gavin Dreyer

**Affiliations:** 1 Department of Paediatrics and Child health, Queen Elizabeth Central Hospital, Blantyre, Malawi; 2 Department of Paediatrics and Child health, University of Cape Town, Cape Town, South Africa; 3 Institute of Immunology and Immunotherapy, University of Birmingham, Birmingham, UK; 4 Department of Nephrology, Birmingham Children’s Hospital, Birmingham, UK; 5 Renal Department, Barts Health NHS Trust, London, UK

**Keywords:** Paediatric kidney disease, Malawi, Outcome

## Abstract

**Background:**

There is a paucity of data on paediatric kidney disease in developing countries such as Malawi. Descriptive research on kidney disease is essential to improving patient outcomes.

**Methods:**

We conducted a cross-sectional study at a tertiary hospital in Malawi from 2012 to 2013. Children under 14 years with proteinuric kidney disease were enrolled from paediatric wards and outpatient clinics at Queen Elizabeth Central Hospital (QECH). Demographic, clinical and laboratory data were collected from patients at enrolment and at 3 months review at which point clinical status and disease outcome were ascertained.

**Results:**

Thirty-four (22 male) patients were studied, mean age 8.54 (SD = 3.62 years). Glomerular disease (*n* = 25, 68%) was the most common presumed renal lesion at presentation. Nephritic syndrome (10) was characterised by a lower baseline complement C3 than nephrotic syndrome (*p* = 0.0027). Seven (47%) cases of nephrotic syndrome achieved complete remission. Eight (80%) cases of nephritic syndrome improved with supportive therapy. Nineteen (56%) patients presented with clinically significant renal damage with eGFR< 60 ml/min/1.73m^2^. Six patients presented in chronic kidney disease (CKD) stage 5 of unclear aetiology, five (83%) died. Three (9%) patients had impaired kidney function and obstructive uropathy demonstrated on ultrasound, two recovered after surgery and one died. Eight (24%) patients had acute kidney injury (AKI) due to primary kidney disease, three of these patients progressed to CKD stage G3a. Seven (21%) patients were lost to follow up.

**Conclusion:**

Kidney disease is a significant cause of mortality and morbidity in children at QECH. Less than half of Nephrotic syndrome cases achieved complete remission. Mortality is highest in children with CKD of unclear cause. Some patients with AKI secondary to primary renal disease progressed to CKD. Understanding the aetiology of paediatric kidney disease and improving patient outcomes by developing enhanced diagnostic and clinical services are priorities at QECH and within Malawi.

## Background

Kidney disease is a cause of significant morbidity and mortality worldwide and the prevalence is rapidly increasing globally [1]. Data from renal registries in developed countries shows that Congenital Anomalies of the Kidney and Urinary Tract (CAKUT) predominate over glomerular disease in children [2, 3]. Renal registries are generally unavailable in developing countries [4]. However, studies done in Africa, Asia, Middle East and South American developing countries all show similar findings [5–7] where in contrast to high income settings, glomerular disease is the predominant cause of paediatric kidney disease.. There is a high prevalence of infectious diseases in developing countries which is a risk factor for glomerular kidney disease [6–9].

The pattern of kidney disease varies in different geographical locations depending on ethnic backgrounds and environmental factors [9]. However, the pattern of kidney disease in children has not been widely studied in Sub Saharan Africa, particularly Malawi and neighbouring countries [10]. While the pattern of kidney disease in Malawi might be similar to other regions in the developing world, the local environment might also lead to a unique pattern of disease.

Detailed research on the aetiology and outcome of paediatric kidney disease is critical to the development of awareness among clinicians and policy makers and will optimize patient outcomes and enable appropriate resource allocation at QECH and nationally. Accordingly, we described kidney disease in children with significant proteinuria at Queen Elizabeth Central Hospital, Blantyre, Malawi.

## Methods

### Study design

We conducted a cross-sectional study with a prospective follow-up period of 3 months at the QECH paediatric department. The primary aim of this study was to describe the clinical presentation and outcome of proteinuric kidney disease in children presenting to a national referral hospital in Malawi.

Enrolment was conducted over a 9-month study period from June 2012 to March 2013, including a seasonal period of high malaria and gastroenteritis incidence. Patients were followed up at a routine paediatric renal clinic at 3 months and outcome determined.

Transport money was provided for the patient and one guardian to assure clinic attendance at 3 months.

### Study site

QECH is a tertiary level facility that serves as the main national referral centre for more than 10 districts in the southern region.

The paediatric department provides free at the point of access primary, secondary and tertiary care for neonates and paediatric patients, seeing approximately 80,000 children and admitting 25,000 children per year.

### Study population

All children less than 14 years with suspected kidney disease were recruited. Patients were referred to the study team if there was clinical suspicion of kidney disease by the admitting paediatric team. This included history, examination and laboratory findings. These patients were either admitted to QECH or were outpatients at the QECH renal clinic. We enrolled all children with the following: significant proteinuria≥2+ (100-300 mg/dl) on urine dipsticks [11] with or without abnormal estimated GFR (eGFR). Patients were excluded if they: declined informed consent or were unwilling to participate; were unwilling or unable to receive clinical care for kidney disease; had isolated urological abnormality (hydrocoele, undescended testes); obstructive uropathy with no proteinuria [12, 13].

### Data collection

We collected patient demographic data combined with a detailed clinical history and examination undertaken by the study team (ZM).

Basic biochemical analysis was undertaken at QECH main laboratory. Serum urea, creatinine, potassium, sodium, chloride and albumin were performed using photometric method on Mindray BS-120 chemistry analyser (Mindray, China). Full Blood Count was conducted on SYSMEX KX-21 N (Sysmex, United States of America). Hepatitis B surface antigen test (SD Bioline, India) and Hepatitis C antibody tests (SD Bioline, India) were conducted using rapid test strips as per manufacturer’s instructions. HIV rapid test were conducted using Determine test strips (Determine, Japan) and Unigold (Trinity Biotech, USA) test strips according to manufacturer’s instructions.

Paracheck plasmodium falciparum (parachekpf) rapid diagnostic test (Orchid Biomedical system, India) was used for malaria screening in all children with fever. Malaria microscopy was conducted in all children with positive malaria rapid diagnostic test.

Immunological assays were performed at the Immunology Department, University of Birmingham, UK. The initial ASOT assays were conducted using Rheumajet ASO latex agglutination (Biokit, Spain) and then repeated on a SPA+ analyser (Binding Site, United Kingdom). IgG, IgA, IgM, C3, C4, Creatinine, CRP, Albumin and Total protein were all measured on Cobas c501 turbidimeter (Roche, Switzerland).

Siemens urine dipstick tests (Siemens, Germany) were used for routine urinalysis. Urine microscopy was conducted at the main QECH Laboratory. The remaining urine specimen was used for urine schistosoma antigen test (Rapid medical diagnostics, South Africa).

Renal ultrasound was conducted by a sonographer using a Phillips HD3 machine (Phillips, Netherlands) using a 3.5 MHz probe. We measured kidney size using length in centimetres and plotted the result against the participant’s age. Kidney size was classified as large (> + 2 SD), small (< − 2 SD) and normal (-2SD to +2SD) for age [9].

### Clinical diagnosis and outcome

The final clinical diagnosis based on standard case definitions was determined at the end of the follow up period for those patients that completed the study (GD, ZM, DM).

#### Nutritional status

We classified nutritional status according to World Health Organisation (WHO) criteria [14] using Z scores to categorise the different anthropometric findings.

#### Renal function

Estimated GFR (eGFR) was calculated using the revised Schwartz formula [15]:

$$ \left(\mathrm{eGFR}\right)={0.413}^{\ast}\mathrm{Height}\ \left(\mathrm{cm}\right)/\mathrm{Plasma}\  \mathrm{Creatinine}\ \left(\mathrm{mg}/\mathrm{dl}\right) $$

#### Hypertension

Blood pressure was classified according to the Fourth report by the United States Working Group on Children and Adolescents of the National High Blood Pressure Education Program (NHBPEP) [16].

#### Diagnostic criteria

Patients were classified as nephrotic syndrome if they had oedema, proteinuria ≥3+ (300-2000 mg/dl) on urine dipstick and hypoalbuminaemia (serum albumin≤25 g/L) [11]. Patients were classified as nephritic syndrome if the presented with oedema, proteinuria ≥2+ (100-300 mg/dl) on urine dipstick, haematuria ≥1+ (5 to 10 red blood cells), hypertension and high creatinine for age.

Acute kidney injury (AKI), where present, was considered a secondary diagnosis due to the primary renal disease. AKI was diagnosed as defined by 2012 KDIGO AKI clinical practice guidelines [17]. We reviewed the eGFR at baseline and at 3 months follow up. Resolution of decreased eGFR at the 3 month follow up was classified as AKI.

Chronic kidney disease (CKD) was defined by KDIGO criteria [18]. Chronicity was based on duration of symptoms or presumed due to findings of small kidneys on KUB ultrasound and low eGFR.

Obstructive uropathy of varying aetiology with evidence of kidney damage was defined as: hydronephrosis, hydroureter, bladder wall thickening on renal ultrasound scan and low eGFR for age.

The clinical and biochemical outcome of study patients was determined at 3 months.We recorded mortality during admission or during follow up period. We classified the clinical status of patients as clinical deterioration, clinical improvement and disease remission according to the clinical diagnosis.

### Clinical care

We provided routine clinical care to all study patients according to local practice guidelines at QECH. Peritoneal dialysis, haemodialysis and kidney biopsies were not available to children in Malawi at the time of this study.

### Statistical analysis

Data analysis was conducted using STATA 2014 (www.stata.com/stata14). The mean and standard deviation were calculated for normally distributed data and the median and inter-quartile range were determined for non-parametric data. Statistical tests of significance were applied to compare different groups. The chi-square test for independence was conducted to compare categorical variables.The Wilcoxon rank sum test was conducted for non-parametric continuous variables to compare two groups. Two sample t-test for independent samples was conducted for parametric data. In order to compare multiple groups of parametric continuous data, we conducted a one way analysis of variance (ANOVA). We conducted a paired t test to determine any differences between paired observations at baseline and 3 months. A *p* value < 0.05 was considered statistically significant.

## Results

Forty two participants with suspected kidney disease were screened, 35 participants with confirmed proteinuric kidney disease were enrolled, 1 withdrew consent. Twenty two (65%) children were male, the mean age 8.54 (SD = 3.62 years). Seventeen (50%) patients were rural dwellers, defined as those residing at least 10 km outside Blantyre urban area.

### Clinical presentation

There was a reported history of recent infection in the preceding 2 weeks in 14 (41%) patients. This included history of sore throat, cough and skin rash. There was recent history of consumption of traditional herbal medicines in 8 (24%) patients. Patients presented to hospital at a median of 14 days (range 3–90 days) after the onset of symptoms. Twenty-eight (82%) patients had microscopic haematuria on urine dipstick. Eight (24%) patients had moderate to severe anaemia (haemoglobin ≤9 g/dl). Nineteen (56%) patients presented with clinically significant renal impairment with eGFR< 60 ml/min/1.73m^2^.

### The role of infections and inflammation

Six (18%) patients were positive for Falciparum malaria on serology or microscopy. Nine (26%) patients were positive for urine schistosoma antigen (Table 1). Schistosoma infection was more common in patients with nephrotic syndrome although there was no statistical significance (*p* = 0.55). ASOT levels at presentation were significantly higher in nephritic syndrome compared to the other conditions (*p* = 0.009). Twenty seven patients tested negative for Hepatitis B and Hepatitis C viral serology, 7 had unknown status.

**Table 1:** Clinical characteristics of patients at presentation and 3 month follow up. Figures are presented as Number (%), mean (SD) and median (IQR)

Parameter (Reference range)	All Patients	Nephrotic Syndrome	Nephritic Syndrome	CKD	Obstructive Uropathy
	(*n* = 34)	(*n* = 15)	(*n* = 10)	(*n* = 6)	(*n* = 3)
Age, years	8.5 (3.6)	8.4 (3.8)	7.9 (2.6)	11.4 (1.5)	6.0 (6.6)
Gender					
Male	22 (65%)	7 (47%)	8(80%)	4 (67%)	3 (100%)
Female	12 (35%)	8 (53%)	2 (20%)	2 (33%)	0 (0%)
HIV status					
HIV negative	30 (88%)	13 (87%)	9 (90%)	5 (83%)	3 (100%)
HIV unknown	4 (12%)	2 (13%)	1 (10%)	1(17%)	0 (0%)
Albumin (35-50 mg/dl)					
Baseline	21 (13–29)	13 (8–16)	25 (22–28)	25 (22–27)	30 (27–33)
3 months	33 (21–44)	36 (18–43)	38 (30–39)	31 (0)	39 (33–45)
Creatinine^a^ (0.2–1.1 mg/dl)					
Baseline	0.9 (0.6–2.8)	0.6 (0.3–1.5)	0.8 (0.6–1.7)	8.0 (7.1–11.9)	2.8 (0.86–3.7)
3 months	0.8 (0.3–1.0)	0.9 (0.2–1.0)	0.6 (0.3–1.0)	10.5 (0)	0.7 (0.6–1.4)
eGFR^a^(65–135 ml/min/1.73m^2^)					
Baseline	47 (26–108)	100 (47–175)	41 (33–95)	6 (4–9)	29 (10–46)
3 months	76 (56–220)	95 (73–275)	116 (49–164)	–	57 (56–67)
Hypertension	19 (56%)	7 (47%)	7 (70%)	4 (67%)	1 (33%)
Schistosoma antigen positive	9 (26%)	5 (33%)	1 (18%)	2 (33%)	1 (33%)
Malaria positive	6 (18%)	0 (0%)	3 (27%)	3 (50%)	0 (0%)

*CKD* Chronic Kidney Disease unclear cause Malaria positive, *RDT* or microscopy, *eGFR* estimated glomerular filtration rate, *Hb* Haemoglobin

^a^Age and sex dependent

### Nephrotic and nephritic syndromes

Glomerular disease (*n* = 25, 74%) was the most common presumed renal lesion (Table 1). Fifteen (44%) patients presented with nephrotic syndrome, seven of these patients had hypertension and/or microscopic haematuria at presentation as well. Patients with nephrotic syndrome presented with severe hypoabuminaemia (*p* < 0.001) and low serum IgG (*p* = 0.002) compared to the other groups. Ten (29%) patients had nephritic syndrome, characterised by a lower C3 (*p* = 0.003) at baseline than those with nephrotic syndrome; C4 levels were normal. ASOT levels were significantly raised at baseline (*p* = 0.0012) in nepritic syndrome compared to nephrotic syndrome (Table 2), consistent with an infective aetiology.

**Table 2:** Comparison of biochemical and immunological data in cases of nephrotic and nephritic syndrome. Figures are presented as mean (SD), median (IQR) and number (%)

Parameter (reference range)	Nephrotic syndrome	Nephritic syndrome	*p*-value
	(*n* = 15)	(*n* = 10)	
Age, years	8.4 (3.8)	7.9 (2.6)	0.61
HIV status			
HIV negative	13 (87%)	9 (90%)	
HIV unknown	2 (13%)	1 (10%)	
Albumin (35–50 mg/dl)			
Baseline	13 (8–16)	25 (22–28)	< 0.001
3 months	36 (18–43)	38 (30–39)	0.26
Creatinine(0.2–1.1 mg/dl)^a^			
Baseline	0.6 (0.3–1.5)	0.8 (0.6–1.7)	0.22
3 months	0.9 (0.2–1.0)	0.6 (0.3–1.0)	0.79
eGFR (65–135 ml/min/1.73m^2^)^a^			
Baseline	100 (47–175)	40 (34–95)	0.95
3 months	95 (73–275)	116 (49–164)	0.83
Complement C3 (0.88–2.52 g/L)			
Baseline	1.9 (1.5–2.5)	0.87 (0.4–1.6)	0.0027
3 months	1.6 (1.5–1.7)	1.7 (1.4–2.0)	0.23
Complement C4(0.12–0.75 g/L)			
Baseline	0.5 (0.3–0.6)	0.31 (0.26–0.37)	0.96
3 months	0.3 (0.3–0.5)	0.31 (0.25–0.35)	0.54
CRP (0-10 mg/dl)			
Baseline	6.7 (2.4–25.9)	4.9 (0.7–16.5)	0.99
3 months	0.3 (0–0.8)	1.3 (0.4–4.1)	0.99
ASOT (0-200 IU/ml)			
Baseline	68 (19–78)	206 (131–528)	0.0012
3 months	70 (60–118)	163 (101–206)	0.0143
IgA (0.2–3.58 g/L)			
Baseline	1.3 (1.0–1.8)	1.50 (1.3–1.7)	0.30
3 months	1.0 (0.9–1.5)	1.46 (1.3–1.6)	0.09
IgM (0.19–2.39 g/L)			
Baseline	1.4 (1.3–1.7)	1.3 (1.1–1.7)	0.69
3 months	1.1 (1.0–1.6)	1.25 (1,0–1.6)	0.52
IgG (4.5–17.1 g/L)			
Baseline	5.4 (4.4–12.9)	15.9 (12.4–19.1)	0.007
3 months	10.3 (10.1–11.7)	16.4 (13.8–19)	0.0006

*Ig* Immunoglobulin, *ASOT* Antistreptolysin O titre, *CRP* C-Reactive Protein, *eGFR* estimated Glomerular filtration rate

^a^Age and sex dependent

Eight cases of nephritic syndrome showed improvement after supportive therapy with anti-hypertensive therapy, diuretics and dietary restriction; 2 patients were lost to follow-up. Seven cases of nephrotic syndrome achieved complete remission after treatment with steroids, 2 showed no remission, 1 died due to sepsis and 5 had no follow up data (Table 3).

**Table 3:** Clinical outcome at 3 months follow up. Figures are presented as number (%)

Outcome^a^	All Patients	Nephrotic syndrome	Nephritic syndrome	CKD	Obstructive uropathy
Vital status	(*n* = 34)	(*n* = 15)	(*n* = 10)	(*n* = 6)	(*n* = 3)
Alive	20 (59%)	9 (60%)	8 (80%)	1 (17%)	2 (67%)
Died	7 (21%)	1 (6.7%)	0	5 (83%)	1 (33%)
Completed study	27 (79%)	10 (63%)	8 (80%)	6 (100%)	3 (100%)
Alive at end of study					
Improvement	17 (85%)	7 (78%)	8 (100%)	0	2 (67%)
Deterioration	3 (15%)	2 (22%)	0	1 (100%)	0

*CKD* Chronic kidney disease

^a^Outcome as defined in methods

### Chronic kidney disease of unclear cause

Six (18%) patients presented in CKD stage 5 (Table 2) of unclear cause. The median days between symptoms and presentation were 36 days. These patients presented with a much lower estimated GFR (eGFR) than other groups (*p* < 0.001). Four of these patients had small kidneys on renal ultrasound scan. All patients had significant proteinuria (> 2+) and microscopic haematuria on urine dipstick suggesting a chronic glomerulonephritis as the underlying aetiology. Five patients died during the study and one had clinical deterioration evidenced by a declining eGFR to lower than 60 ml/min/1.73m^2^ (Table 3). None of these patients received renal replacement therapy. The aetiology of renal disease was unclear in all these patients and none had a kidney biopsy.

### Obstructive Uropathy

Three (9%) patients had evidence of urinary tract obstruction. All patients had significant impairment of kidney function with eGFR< 60 ml/min/1.73m^2^ and significant proteinuria. Two of these patients were diagnosed to have posterior urethral valves on cystoscopy and micturating cysto-urethrogram (MCUG), the third patient had bladder outlet obstruction secondary to urinary bladder mass. The latter patient died from complications of kidney failure, whilst the other two patients showed clinical improvement in GFR after valve ablation.

### Acute kidney injury

Eight (24%) patients had acute kidney injury due to the various primary kidney diseases. Two nephritic syndrome, 3 nephrotic syndrome and 3 had obstructive uropathy. All patients had AKI stage 3 (eGFR< 35 ml/min/1.73m^2^ or serum Creatinine > 4.0 mg/dl). All patients showed improvement in eGFR at 3 months. At follow up, 5 patients had an eGFR above 60 ml/min/1.73m^2^, one had normalization of eGFR above 90 ml/min/1.73m^2^. Three patients had improvement of eGFR but remained lower than 60 ml/min/1.73m^2^ (mean eGFR 54 ml/min/1.73m^2)^, consistent with CKD stage G3a after an episode of AKI.

### Clinical outcomes

The clinical outcome of each condition is presented in Table 3. CKD stage G4 to G5 carried the worst prognosis, whereas nephritic syndrome had the most favourable outcome. Seven (21%) patients were lost to follow up, five with nephrotic syndrome.

## Discussion

To the best of our knowledge, this is the first study in Malawi and the sub-region to present detailed clinical and immunological data on proteinuric kidney disease in a paediatric population.

Glomerular disease was the predominant cause of kidney disease, similar to findings in multiple studies in Nigerian tertiary facilities [19–24]. There was a clear immunological delineation between cases of nephrotic and nephritic syndromes. Nephritic syndrome biochemistry was consistent with a post infectious aetiology suggested by significantly high ASOT and low C3 at presentation, consistent with a post infectious glomerulonephritis [25, 26]; C3 that increased and normalised at follow up.

In this study, 47% of patients with nephrotic syndrome were steroid responsive. Steroid sensitivity of nephrotic syndrome in black African children seems to vary, while previous studies reported low rates, current studies have shown response rates as high as 75.9% in some parts of Nigeria [27]. Seven of the 15 patients with nephrotic syndrome presented with unusual findings: they all had hypertension, six had persistent microscopic haematuria and four had renal insufficiency. These patients present a diagnostic challenge in the absence of kidney histopathology. However, the classification of nephrotic syndrome was due to no history of antecedent infection and low ASOT level, no frank haematuria and normal complement C3 levels. These findings may be consistent with the clinical phenotype of childhood focal segmental glomerulosclerosis (FSGS). The identification of this population is very important due to the poor outcomes associated with FSGS in black patients of African descent [28] who require more aggressive treatment in order to mitigate the risk of progression to CKD and mortality. Additionally, a significant proportion of our patients presented with advanced kidney damage of unclear aetiology. Glomerular disease was suggested by the presence of significant proteinuria and microscopic haematuria. Chronicity of disease was based on the finding of small kidneys in four of the six patients on renal ultrasound scan. This suggests previously undiagnosed or sub-optimally managed glomerulonephritis. Patients with CKD stage G5 had the worst outcome due to the unavailability of renal replacement therapy as a therapeutic option during the time of the study.

A significant proportion of patients in our study presented with AKI as a consequence of an underlying primary renal lesion. Acute kidney injury has been shown to significantly increase the risk of developing chronic kidney disease and progression to ESKD [29] which in a setting without paediatric nephrologists and renal replacement therapy leads to high mortality.

Patients with nephritic syndrome exhibited clinical improvement with supportive care. Three patients with nephritic syndrome and AKI developed CKD, underscoring the risk of progression to CKD in patients with nephritic syndrome and AKI. Obstructive uropathy secondary to posterior urethral valves showed improvement after surgical intervention, highlighting the benefit of specialist evaluation and prompt referral to tertiary surgical services.

The overall outcome in patients in this study is poor. Late presentation to hospital may have also contributed to poor outcome, and low socio-economic status possibly contributed to the high rate of loss to follow up, despite the provision of transport refunds to facilitate clinic attendance. Public transport is unreliable in rural areas and contributes to poor access to health care, highlighting challenges in the provision of optimal health services in a resource limited setting. Furthermore, some patients who originally presented with oedema due to nephrotic syndrome might not have returned once their presenting oedema resolved as the family may have presumed the symptoms would not recur. Lack of awareness of kidney disease and diagnostic limitations in primary and secondary health facilities may have contributed to late presentation which reduces opportunities for early and specialist disease modifying interventions.

Our study also provides a clinical, immunological and biochemical profile of children with proteinuric kidney disease at QECH. Poor socio-economic circumstances are prevalent globally, suggesting our study may be applicable in similar patient populations. Access to the immunological diagnostic investigations we performed cannot easily be sustained in a resource limited setting, reaffirming the importance of good clinical history and examination as a diagnostic tool. Our data will add to knowledge of the management of paediatric kidney disease and outlines its poor outcomes in some groups but also the potential positive benefit that conservative care with accessible interventions in Malawi and similar settings can provide, especially in patients with glomerular lesions.

Our study is limited due to recruitment occurring during office hours only since we were only able to include referred cases rather than conducting active case finding for renal disease across the whole of the in-patient service. Thus, there is a degree of selection bias in our cohort and the small sample size makes it difficult to ascertain the true prevalence of paediatric proteinuric kidney disease presenting to QECH. The study was conducted at a tertiary hospital and thus our cohort is possibly not representative of kidney disease in district hospitals in Malawi. We also recognise that the high loss to follow up may also lead to bias. Finally, the lack of kidney histopathology prevents a more detailed understanding of the specific glomerular pathology in our cohort.

There is a pressing need for further research into paediatric kidney disease in Malawi in order to raise awareness of the local prevailing phenotype of paediatric kidney disease and improve patient outcomes. Supporting our study, a larger study of AKI at QECH is currently underway. Glomerular disease as the primary aetiology in our study would be better assessed with renal histology but this service for logistical, technical and safety reasons is unlikely to be available in the foreseeable future.

## Conclusion

Paediatric proteinuric kidney disease is a cause of significant morbidity and mortality at QECH, and acute kidney injury has been shown to cause CKD in some children. A significant proportion of patients presented in CKD of unclear cause in a setting without renal replacement therapy for children, with resulting high mortality. However, many common paediatric renal conditions (nephritic/nephritic syndrome) are amenable to treatment with simple, conservative and surgical measures available at QECH and elsewhere. Histolopathological characterisation of proteinuric kidney disease in this region will lead to greater understanding of aetiology.

Raising awareness in developing countries is essential to early identification and treatment of kidney disease.

## Abbreviations

AKI: Acute kidney injury
ASOT: Antistreptolysin O titre
CAKUT: Congenital anomalies of the kidney and urinary tract
CKD: Chronic kidney disease
CRP: C-reactive protein
eGFR: Estimated glomerular filtration rate
ESKD: End stage kidney disease
FSGS: Focal segmental glomerulosclerosis
IgA: Immunoglobulin A
IgG: Immunoglobulin G
IgM: Immunoglobulin M
MCUG: Micturating cystourethrogram
NHBPEP: National High Blood Pressure Education Program
WHO: World Health Organisation

## References

[1] Liyanage T, Ninomiya T, Jha V, Neal B, Patrice HM, Okpechi I (2015). Worldwide access to treatment for end-stage kidney disease: a systematic review. Lancet.

[2] Ardissino G, Dacco V, Testa S, Bonaudo R, Claris-Appiani A, Taioli E (2003). Epidemiology of chronic renal failure in children: data from the ItalKid project. Pediatrics.

[3] Pistor K, Scharer K, Olbing H, Tamminen-Mobius T (1985). Children with chronic renal failure in the Federal Republic of Germany: II. Primary renal diseases, age and intervals from early renal failure to renal death. Arbeitsgemeinschaft fur Padiatrische Nephrologie. Clin Nephrol.

[4] Liu F, Rutherford P, Smoyer-Tomic K, Prichard S, Laplante S. A global overview of renal registries: a systematic review. BMC Nephrol. 2015;16(31):4. 10.1186/s12882-015-0028-2 PMC4377012 25886028

[5] Ladapo TA, Esozobor CI, Lesi FE (2014). Pediatric kidney diseases in an African country: prevalence, spectrum and outcome. Saudi J Kidney Dis Transpl.

[6] Yadav SP, Shah GS, Mishra OP, Baral N (2016). Pattern of renal diseases in children: a developing country experience. Saudi J Kidney Dis Transpl.

[7] Ali SH, Hussein FS, Abd Al-Amer H (2015). Profile of renal diseases in Iraqi children: a single-center report. Saudi J Kidney Dis Transpl.

[8] Kayange NM, Smart LR, Downs JA, Maskini M, Fitzgerald DW, Peck RN. The influence of HIV and schistosomiasis on renal function: a cross-sectional study among children at a hospital in Tanzania. PLoS Negl Trop Dis. 2015;9(1):5-8. 10.1371/journal.pntd.0003472 PMC4303314 25612312

[9] Kayange NM, Smart LR, Tallman JE, Chu EY, Fitzgerald DW, Pain KJ, Peck RN (2015). Kidney disease among children in sub-Saharan Africa: systematic review. Pediatr Res.

[10] Stanifer JW, Jing B, Tolan S, Helmke N, Mukerjee R, Naicker S (2014). The epidemiology of chronic kidney disease in sub-Saharan Africa: a systematic review and meta-analysis. Lancet Glob Health.

[11] Kidney Disease (2012). Improving global outcomes (KDIGO) Glomerulonephritis work group. Clinical practice guideline for glomerulonephritis. Kidney Inter Suppl.

[12] Churg J, Habib R, White RH (1970). Pathology of the nephrotic syndrome in children: a report for the international study of kidney disease in children. Lancet.

[13] Rosenbaum DM, Korngold E, Teele RL (1984). Sonographic assessment of renal length in normal children. Am J Radiol.

[14] World health organisation. WHO child growth standards and the identification of severe acute malnutrition in infants and children: a joint statement by the World Health Organization and the United Nations Children’s fund. Geneva: WHO Press; 2009. 24809116

[15] Schwartz GJ, Muñoz A, Schneider MF, Mak RH, Kaskel F, Warady BA (2009). New equations to estimate GFR in children with CKD. J Am Soc Nephrol.

[16] National High Blood Pressure Education Program Working Group on High Blood Pressure in Children and Adolescents (2004). The fourth report on the diagnosis, evaluation, and treatment of high blood pressure in children and adolescents. Pediatrics.

[17] Kidney Disease (2012). Improving global outcomes (KDIGO) acute kidney injury work group. KDIGO clinical practice guideline for acute kidney injury. Kidney Inter.

[18] Kidney Disease (2013). Improving global outcomes (KDIGO) CKD work group. 2012 clinical practice guideline for the evaluation and Management of Chronic Kidney Disease. Kidney Int Suppl.

[19] Esezobor CI, Ladapo TA, Osinaike B, Lesi FE (2012). Paediatric acute kidney injury in a tertiary hospital in Nigeria: prevalence, causes and mortality rate. PLoS One.

[20] Okoro BA, Okafor HU (1999). Pattern of childhood renal disorders in Enugu. Niger J Paediatr.

[21] Etuk IS, Anah MU, Ochighs SO, Eyong M (2006). Pattern of pediatric renal disease in inpatients in Calabar, Nigeria. Trop Dr.

[22] Onifade EU (2003). A 10-year review of childhood renal admissions into the Lagos University teaching hospital, Nigeria. Nig Q J Hosp Med.

[23] Michael IO, Gabriel OE (2003). Pattern of renal diseases in children in Midwestern zone of Nigeria. Saudi J Kidney Dis Transplant.

[24] Ocheke IE, Okolo SN, Bode-Thomas F (2010). Pattern of childhood renal diseases in Jos, Nigeria: a Pleminary report. J Med Trop.

[25] Ramdani B, Zamd M, Hachim K, Soulami M, Ezzahidy M, Souiri M (2012). Acute post infectious glomerulonephritis. NephrolTher.

[26] Gunasekaran K, Krishnamurthy S, Mahadevan S, Harish BN, Kumar AP (2015). Clinical characteristics and outcome of post-infectious Glomerulonephritis in children in southern India: a prospective study. Indian J Pediatr.

[27] Ladapo TA, Esozobor CI, Lesi FE (2014). High steroid sensitivity among children with Nephrotic syndrome in southwestern Nigeria. Int J Nephrol.

[28] Korbet SM, Wilcox CS, Brady HR (2003). Primary focal segmental glomerulosclerosis. Therapy in nephrology and hypertension.

[29] Coca SG, Singanamala S, Parikh CR (2012). Chronic kidney disease after acute kidney injury: a systematic review and meta-analysis. Kidney Int.

